# A Conceptual Review of Naturally Occurring Toxins and Venoms as Peptide Blockers to Combat Chronic Low Back Pain

**DOI:** 10.1002/jsp2.70107

**Published:** 2025-08-15

**Authors:** James Melrose, Stone Sima, Neha Chopra, Ashish Diwan, Zi Gu

**Affiliations:** ^1^ Raymond Purves Bone and Joint Research Laboratory Kolling Institute, Sydney University Faculty of Medicine and Health, Northern Sydney Area Health District, Royal North Shore Hospital St. Leonards New South Wales Australia; ^2^ Graduate School of Biomedical Engineering The University of New South Wales Kensington, Sydney New South Wales Australia; ^3^ Spine Labs, Department of Orthopaedic Surgery, St. George & Sutherland Clinical Campuses, School of Clinical Medicine, Faculty of Medicine and Health University of New South Wales Kogarah, Sydney New South Wales Australia; ^4^ Spine Service, Department of Orthopaedic Surgery, St. George & Sutherland Clinical Campuses, School of Clinical Medicine, Faculty of Medicine and Health University of New South Wales Kogarah, Sydney New South Wales Australia; ^5^ Spinal Unit, Discipline of Orthopaedic Surgery, School of Medicine The University of Adelaide & The Royal Adelaide Hospital North Terrace, Adelaide South Australia Australia; ^6^ NanoBiotechnology Research Laboratory, School of Chemical Engineering University of New South Wales Kensington, Sydney New South Wales Australia

**Keywords:** blocking of voltage gated Ca^2+^ channels, conotoxins, dorsal root ganglion, intervertebral disc degeneration, low back pain, nerve‐blocks, neuroinflammation, nigriventer venom peptides, nociception

## Abstract

**Background:**

One of the significant putative causes of low back pain (LBP) is degeneration of the intervertebral disc (IVD). Degenerated discs exhibit loss of proteoglycans, notably aggrecan, leading to mechanical dysfunction and aberrant nerve ingrowth. This pathological innervation results in the proliferation of nociceptive and mechanoreceptive neurons, significantly contributing to persistent pain. A critical therapeutic target is the dorsal root ganglion (DRG), which serves as a key neural hub for nociceptive signaling and neurogenic inflammation. Increased calcium influx through voltage‐gated calcium channels within DRG neurons underpins heightened neuronal excitability, facilitating persistent pain transmission. Recent evidence highlights the promising role of bioactive peptides derived from reptilian and insect venoms as potent calcium channel blockers.

**Methods:**

This conceptual review explores published evidence and mechanistic rationale on naturally occurring toxins and venoms as peptide calcium channel blockers for chronic LBP. We considered DRG targeted mechanisms and delivery approaches, including incorporation into biomimetic proteoglycans for localized, sustained intradiscal release, and their use along conventional nerve block procedures.

**Results:**

Venom derived peptide families including ω‐conotoxins from cone snail and Tx3‐family spider peptides from Phoneutria nigriventer selectively block neuronal calcium channels (notably Ca_v_2.2), thereby reducing the release of neurotransmitters that propogate pain signals. Alongside these antinocicpetive effects, the targeted mechanism of action and directed modalities of these peptides offer a novel therapeutic approach with potential advantages over tradiitonal analgesics, which often present challenges related to tolerance and systemic side effects.

**Conclusion:**

Naturally occurring bioactive peptide calcium channel blockers delivered either directly to the DRG or through a multifaceted therapeutic approach with biomimetic proteoglycans into the IVD or conventional nerve block procedures into the epidural space resents a promising future direction in managing chronic LBP. This approach warrants further pre‐clinical and clinical evaluation to clarify clinical utility, potentially transforming pain management paradigms and significantly reducing healthcare burdens associated with chronic spinal disorders.

## Graphical abstract

This review explores the therapeutic potential of naturally occurring venom‐derived peptides, particularly from reptiles and insects, as novel calcium channel blockers for managing chronic low back pain caused by intervertebral disc degeneration. These peptides offer targeted analgesia by inhibiting pathological nerve ingrowth and neurogenic inflammation in degenerated discs, presenting a promising alternative or adjunct to conventional treatments.

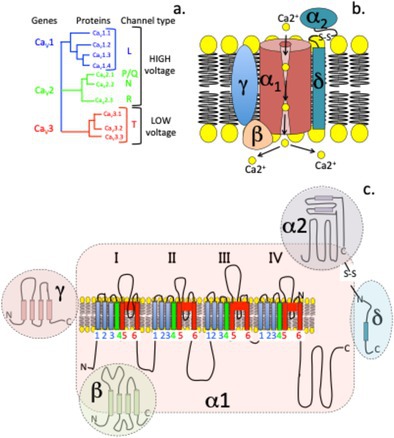

## 1 Introduction

Intervertebral disc degeneration (IVDD) is a major contributor to low back pain (LBP) due to the ingrowth of nerves from the periphery of the IVD into inner AF regions of the degenerate IVD [1]. This leads to a significant increase in mechano‐ and nociceptive neuron numbers in the IVD which produce pain responses upon their sensitization in the biomechanically incompetent degenerate IVD depleted of its weight bearing aggrecan [2]. Multiple anatomical structures in the spine besides the IVD can also contribute to pain responses, including nerves located in the vertebral body, cartilaginous endplate, paradiscal myotendinous tissues, and osteoarthritic facet joint articular cartilage [3]. Degenerative changes in spinal muscles and ligaments also occur in response to IVDD and may also contribute to spinal pain, reduced spinal stability, flexibility, and impaired locomotion [4].

Many in vivo animal models have been used for the measurement of neuromuscular responses following spinal manipulation of the normal spine, in spines that had undergone spinal surgery, and in spinal segments containing degenerate IVDs [5, 6, 7, 8]. This model has proven valuable for understanding the underlying mechanisms of spinal disorders, including the involvement of the dorsal root ganglion (DRG) and calcium (Ca^2+^) channels in nociceptive signaling. Following nerve injury or inflammation, the DRG becomes a key site of increased pain signaling, facilitated by the influx of Ca^2+^ through voltage‐gated channels [9, 10]. This process contributes to heightened neuronal excitation and the release of neurostimulatory peptides, which exacerbate pain responses [11].

Recent studies have shown that many naturally occurring bioactive peptides, particularly those derived from animal venom, exhibit anti‐nociceptive properties by effectively blocking Ca^2+^ channels in the DRG. These peptides hold significant potential for pain management, particularly in conditions involving neurogenic inflammation and nerve ingrowth into degenerate IVDs. The aim of this review is to explore the therapeutic potential of nerve‐blocking agents, specifically targeting the pathological nerve ingrowth and inflammation associated with degenerate IVDs, which directly affect the DRG and contribute to chronic pain conditions.

## 2 The Significant Burden of Chronic LBP

A 10‐year global study of major musculoskeletal disorders has shown LBP is the number one musculoskeletal condition in terms of its impact on disability year duration and its socioeconomic impact [12]. IVDD affects approximately 80% of the general population, and in a subset of these individuals, the resultant LBP is of sufficient severity to warrant treatment by a physician [13] and results in lost work days [14]. LBP cost the UK Health System £12.3 billion [15], and Australian Healthcare System $9.17 billion [16] in 2000–2003. In 2006, LBP cost the United States Healthcare System in excess of $100 billion [17] and in 2016 $134 billion [18].

It has been estimated that ~619 million people are afflicted by LBP globally [19, 20, 21]. Increased LBP incidence in the 5th and 6th decades [22] means that with the advancing age of the global general population [23] the impact of LBP will only increase in the ensuing two decades.

## 3 Pathomechanics of LBP

LBP has been categorized as mechanical, non‐mechanical, or due to referred pain [24, 25]. Neuropathic pain (NeP) is defined as pain resulting from a lesion or disease affecting the somatosensory nervous system. Specifically in the spine, NeP commonly arises from inflammation or irritation of neural tissues. This is distinct from actual or potential damage to nonneural tissue, which can lead to the activation of nociceptors, leading to nociceptive pain (NoP) [24, 26, 27]. Accurate classification of pain based on these underlying mechanisms is crucial for effective patient management [28, 29]. Trauma to the PNS/CNS can lead to pain hypersensitivity, with NeP persisting in some tissues for prolonged periods.

IVD degeneration can lead to both NoP and NeP. Early‐stage degeneration primarily results in NoP due to mechanical stress and microtears in the annulus fibrosus, activating nociceptors. As degeneration progresses, however, it can trigger NeP through the release of pro‐inflammatory cytokines and neurotrophic factors, leading to nerve damage and heightened pain sensitivity [30, 31]. In severe cases, herniation of the degenerated disc can mechanically compress nerve roots, further exacerbating NeP [32]. Recognizing the multifaceted nature of disk degeneration and its potential to generate both types of pain underscores the need for further research into novel pain management approaches.

## 4 Innervation of the IVD

The IVD is innervated by branches of the sinuvertebral nerve, ventral rami spinal nerves, and by gray rami communicantes [33]. The sinuvertebral nerve is a mixed nerve, combining somatic sensory fibers originating from the DRG and autonomic fibers derived from the sympathetic trunk, which together re‐enter the spinal canal to innervate the posterior annulus fibrosus, posterior longitudinal ligament, and vertebral periosteum. The basivertebral nerve, a key branch arising from the sinuvertebral nerve, travels through the vertebral body and innervates the vertebral endplates. It is important to note that while the nerves transmit sensory input back to the DRG, they do not terminate within the ganglion itself. In the normal healthy IVD, innervation is restricted to the outermost lamella of the AF. Small nociceptive nerve fibers and larger mechanoreceptor fibers are both present. However, with IVDD, the major IVD proteoglycan, aggrecan, becomes degraded; there is a drop in IVD hydration, and hydrostatic pressure results in conditions where an ingrowth of small blood vessels and nerves into the degenerate IVD becomes possible. In the normal loaded IVD, the internal pressure physically prevents the ingrowth of nerves and blood vessels; in addition, active molecular mechanisms, such as repulsive signaling from semaphorins and the relative absence of pro‐innervation and angiogenic signals, further inhibit nerve and vessel ingrowth under healthy conditions [3, 34].

**FIGURE 1:**
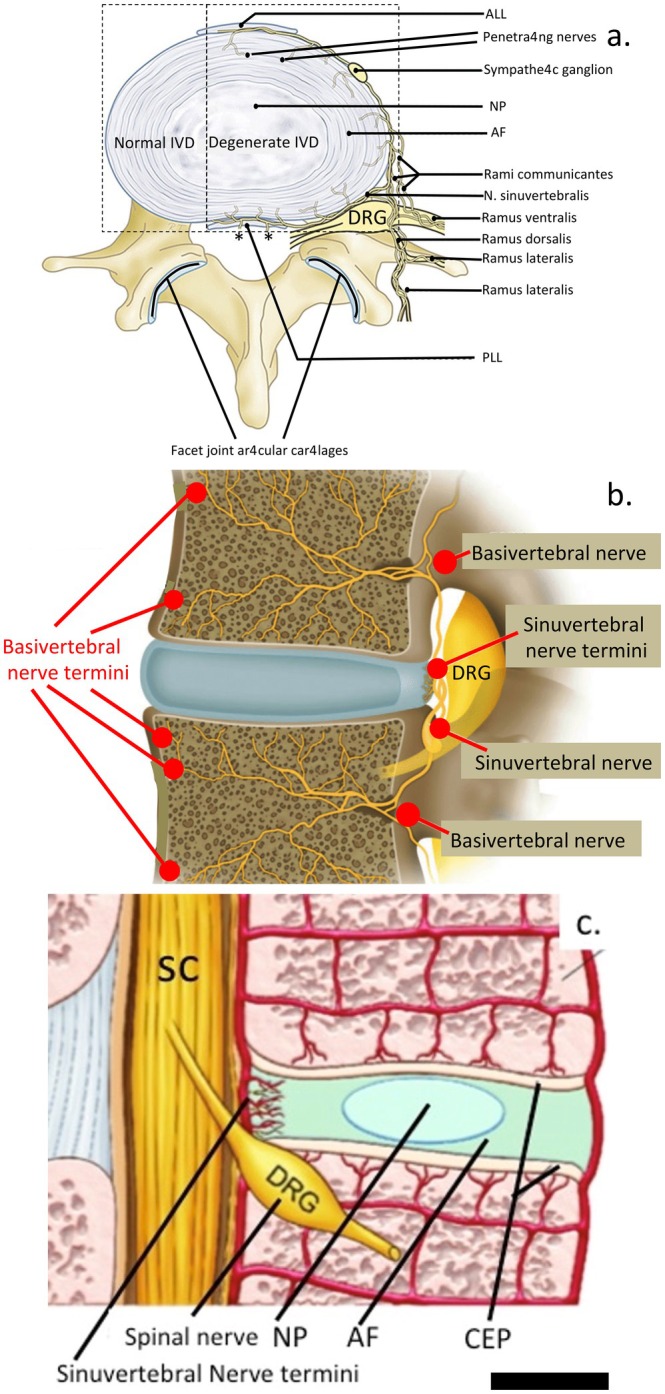
Schematic depiction of the innervation of the degenerate IVD and associated tissues. Figure part (a) reproduced from [3], (b) modified from [35] with permission. ALL, anterior longitudinal ligament; DRG, dorsal root ganglion; PLL, posterior longitudinal ligament; and (c) schematic depiction of the dorsal root ganglion in relation to the IVD and associated spinal tissues, SC, spinal cord.

DRG contain thin myelinated and unmyelinated fibers arising from small nociceptive neurons projecting from the outermost IVD laminae I and II, extending to the dorsal horn of the spinal cord (Figure 1a,b) [27]. Most of these fibers originate from small peptidergic neurons expressing tyrosine kinase receptors TrkA/TrkB [3, 36, 37], while non‐peptidergic neurons express the Ret receptor for glial cell‐derived neurotrophic factor (GDNF) [36]. In addition to neurotrophin receptors, DRG neurons differentially express various channels and receptors, including degenerin/epithelial sodium channels (DEG/ENaCs: ENaCα, β, and γ) [38]; acid‐sensing ion channels (ASIC 1–3); transient receptor potential (TRP) channels (TRPA1, TRPC1, TRPC6, and TRPV1–4); glial cell line‐derived neurotrophic factor receptors α1 and α3 (GFRα1, GFRα3); ATP‐gated ion channel subtype P2X3; and vanilloid receptor subtype 1 (VR1). Additionally, some DRG neurons express neuropeptides such as calcitonin gene‐related peptide (CGRP), thiamine monophosphate (TMP), and substance P. These channels, receptors, and neuropeptides may interact with or modulate the action of neurotrophic factors, contributing to nociceptive signaling.

### 4.1 Nerve Morphology and Ingrowth Into the Degenerate IVD

In healthy discs, sensory and sympathetic fibers are typically limited to the outer layers of the annulus fibrosus and the vertebral endplates, as shown through immunohistochemical analysis [39, 40, 41]. Mechanoreceptors, including Ruffini endings, Pacinian corpuscles, and Golgi tendon organ–like structures, along with sensory neuropeptides such as substance P and calcitonin gene‐related peptide, play important roles in detecting mechanical changes and transmitting pain signals [42, 43]. When degeneration occurs, these fibers penetrate deeper into the inner annulus and even the nucleus pulposus, a process encouraged by increased levels of inflammatory and neurotrophic factors, contributing to heightened nociception and the development of pain [44]. This has allowed the identification of small nociceptive and larger mechanoreceptors in the IVD; however, the nerve cell soma is not within IVD tissues and is present within the DRGs, which communicate with IVDs.

IVDD and discogenic LBP accompany internal disruption in normal IVD architecture and loss of the major IVD PG aggrecan. Lesions that subsequently develop in the degenerate AF are associated with pathological ingrowth of branches of the sinuvertebral nerve, increased numbers of nociceptive nerves, and pain generation [45, 46]. With mechanical overload of the degenerate IVD, disruption in CEP structure can also occur and is a significant contributor to the generation of LBP and an initiator of IVDD [35]. The CEP is densely innervated by the basivertebral branches of the sinuvertebral nerve, and pathological ingrowth of these nerves into the CEP from the vertebral body occurs during IVDD, leading to pain generation. Immunohistochemical studies have identified several key neural markers in the nerves of the CEP, including S100 (a marker of Schwann cells), protein gene product 9.5 (PGP9.5, a pan‐neuronal marker), substance P, and growth‐associated protein 43 (GAP43, a marker of nerve sprouting and regeneration) [45, 46, 47, 48, 49]. Additionally, GAP43, PGP9.5, and glial fibrillary acidic protein have been localized in ingrowing nerves within the degenerated AF [2], where they are associated with vertebral remodeling adjacent to annular defect sites [50]. The detection of these markers reflects ongoing nerve ingrowth, neural remodeling, and an increased nociceptive potential in degenerated disc tissue.

## 5 The Role of the DRG in Pain Generation

Degeneration of an IVD can trigger neurogenic inflammation not only within the affected disc but also in adjacent healthy discs [51]. In a healthy disc, the outer AF is innervated by nociceptive nerve fibers, but during degeneration, these fibers extend into the inner AF and NP, releasing neuropeptides that drive local inflammation. IVDD is closely associated with the production of inflammatory factors that enhance nociceptor sensitization and amplify pain signals [30]. Neurogenic inflammation arises from the release of neuropeptides by activated primary afferent terminals of multisegmental DRG neurons following injury to the AF [52]. This local inflammatory response can also spread to neighboring healthy discs through antidromic activity, where impulses travel in the reverse direction along the axon—away from the nerve terminals and toward the soma—thereby promoting inflammation beyond the initial injury site (Figure 1c). The DRG plays a central role in sensory transduction and neuromodulation, particularly in pain transmission and the persistence of NeP states [53]. Given the heterogeneity of DRG sensory neurons, the analgesic effects of treatments aimed at alleviating IVD‐related pain may result from the modulation of one or several neuronal subtypes within the DRG [54].

### 5.1 Voltage‐Gated Calcium Channels

Deafferentation pain occurs in orthopedic patients as a result of nervous system injury, often arising from spinal cord or peripheral nerve damage and leading to NeP syndromes [55]. A knock‐in mouse model using hemagglutinin‐labeled Ca_V_2.2 has been used to visualize the expression pattern of endogenous Ca_V_2.2 signaling in DRG neurons projecting to primary afferents in the dorsal horn, clearly demonstrating the potential of selective calcium channel–blocking agents for treating deafferentation‐associated IVD pain [56]. Calcium signaling plays a key role in regulating sensory input in NeP, and agents that inhibit this activity represent a logical therapeutic strategy. Following nerve injury or inflammation, the DRG may become a significant source of increased nociceptive signaling due to heightened neuronal excitation and the release of peptides responsible for neurostimulation [9, 10, 11, 57, 58]. This heightened signaling is driven by increased calcium influx through voltage‐gated channels and the mobilization of neurotransmitters stored in synaptic vesicles. Calcium signaling is notably elevated in neurons after spinal nerve injury, highlighting the therapeutic potential of DRG‐targeted anesthesia to prevent the development of pathological pain states [59, 60].

Importantly, the DRG lacks a protective perineurial membrane, making it a permeable and accessible target for drug administration [61]. Peripheral sensory neurons, including those within the DRG, rely primarily on voltage‐gated sodium channels to propagate action potentials, while voltage‐gated calcium channels mediate neurotransmitter release through vesicle exocytosis. Targeting pain pathways with specific calcium channel–blocking agents, as proposed in this study, holds considerable promise for providing more precise mechanisms of action and improving the management of intradiscal pain.

### 5.2 Roles of Potassium and Sodium Channels

Mechano‐sensitive ion channels (MSCs) involved in neuronal signaling include the big potassium channel (BK); two‐pore domain potassium channels (K2Ps) such as TRAAK and TREK; Piezo channels; the epithelial sodium channel (ENaC); and transient receptor potential vanilloid channels TRPV1, TRPV2, and TRPV4 [62]. These channels are activated by various physiological stimuli, including mechanical stress, changes in osmolarity and temperature, physical deformation, and pH. Among these, TRPV1 is a key detector of pain‐producing stimuli. It interacts with several voltage‐gated ion channels, including Nav1.9, Kv4.3, and Ca_v_2.2, particularly in small‐sized DRG neurons [63]. The association of Kvβ subunits with TRPV1 channels promotes their trafficking to the plasma membrane, enhancing their capacity to mediate pain perception [64, 65]. Additionally, Slack channels, encoded by KCNT1, are expressed widely across neurons in the central and peripheral nervous systems [66].

Extracellular sodium ions ([Na+]o) also regulate TRPV1 activity, with studies showing that low [Na+]o can activate TRPV1 in vitro [67]. Voltage‐gated sodium channels (Navs), particularly Nav1.7, are major drivers of sensory neuron excitability and are crucial for pain signal transmission [68]. Nine Nav subtypes (Nav1.1–Nav1.9) have been identified, and compounds targeting these channels may modulate nerve conduction and inhibit TRP channel‐mediated pain, suggesting their therapeutic potential alongside calcium channel blockers for pain relief in the IVD [69].

### 5.3 Targeting the DRG

The DRG has been explored as a target for pain therapies, with interventions such as COX‐2 inhibitors (e.g., celecoxib) [70] and DRG nerve blocks showing efficacy in treating discogenic LBP [71]. This area of clinical research has established proof of principle for biomolecular approaches to chronic LBP management [72]. Importantly, afferent neurons, which transmit sensory information from the IVD to the brain, differ structurally from efferent neurons, which carry motor signals from the central nervous system. Afferent neuron cell bodies reside in the DRG, making the DRG an accessible and promising target for therapies aimed at reducing nociceptive signaling and alleviating discogenic pain [52, 73, 74].

## 6 Prospective Use of Venoms and Toxins to Control Pain Generation

### 6.1 Calcium Signaling in Neurons and Nociceptive Processes

**FIGURE 2:**
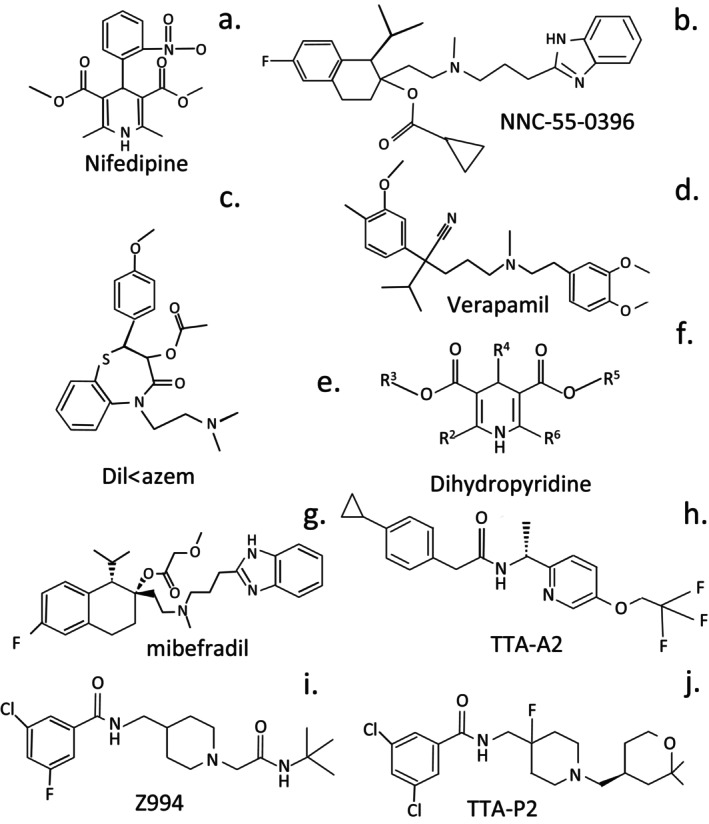
The varied structures of Ca channel blocking compounds that have found application in nerve‐block strategies in different classes of Ca channels in the alleviation of chronic pain.

Calcium has essential roles to play in neural activation and neural transduction. An influx of Ca^2+^ occurs into neurons during neuronal activation and is an essential aspect of the neurotransductive process. During neuronal activation, a wave of membrane depolarization occurs from the soma down the axon to the dendritic tip and synaptic gap; this results in an influx of Ca^2+^ into the neuron through voltage‐gated Ca^2+^ channels [75, 76]. Presynaptic Ca^2+^ influx is a key signal for synaptic vesicle neurotransmitter transport to the synaptic gap and release [77, 78]. Synaptotagmin has been proposed as a Ca^2+^ bio‐sensor that regulates synaptic vesicle transport by the 12‐span transmembrane synaptic vesicle KS transport PG SV2 [79]. The soluble N‐ethylmaleimide‐sensitive factor attachment protein receptor (SNARE) complex (syntaxin‐1, SNAP‐25, and synaptobrevin) regulates membrane fusion of the exported synaptic vesicle with the de‐polarized presynaptic membrane [80]. This results in controlled release of neurotransmitter peptides into the synaptic gap, where they are subsequently taken up by communicating neurons in the neural network during neurotransduction. The SNAP receptor SNARE proteins mediate neurotransmitter release by forming tight complexes fusing synaptic vesicles with the presynaptic plasma membranes in microseconds. This process is regulated by the cytoplasmic neuronal protein, complexin (synaphin 1 and 2) which binds to the SNARE protein complex with a high affinity [81, 82]. In the presence of Ca^2+^, synaptotagmin displaces complexin, allowing the SNARE protein complex to bind the transport vesicle to the presynaptic membrane. Complexin can act both as an inhibitor and a facilitator of synaptic vesicle fusion and neurotransmitter release [81]. Blocking of neuronal Ca signaling by bioactive peptides present in reptile and insect venoms is the basis of their anti‐nociceptive properties in pain alleviation and of therapeutic application in nerve‐blocks, and in our opinion, are worthy of consideration in the development of procedures to control nociception in the degenerate IVD (Figure 2). Furthermore, they could be incorporated into biomimetic proteoglycans (PGs) and administered into degenerate IVDs, where this would act as a pro‐drug delivery system [83].

**FIGURE 3:**
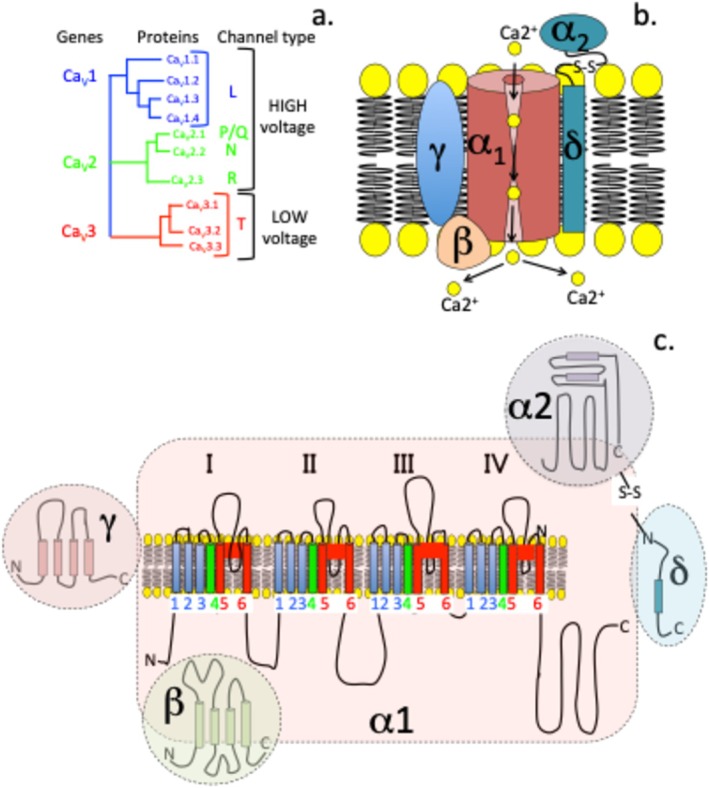
The heteromultimeric structural organization of the transmembrane Ca^2+^ voltage gated channel complex showing (a) the three classes of voltage gated Ca channels and their 10 isoforms which have been categorized into channels responsive to high and low voltages generated by membrane depolarization. (b) Schematic depiction of the structural organization of the voltage gated Ca channel showing the central α1 sub‐unit pore forming Ca channel and auxiliary transmembrane γ, cytoplasmic β and α2δ subunits which can modify the electrophysiological and pharmacological responses in the α1‐sub‐unit. (c) Expanded view of the α1.1‐Ca_V_ sub‐unit and its pore‐forming sub‐structure and auxiliary γ, β, α2δ sub‐units, S1–S6 modules in four homologous α1 sub‐unit domains (I–IV) are shown, S1–S4 are voltage sensing modules, S4 with voltage gating +ve charge is shown in green. S5 and S6 and the P‐loop between them (red) have pore forming roles. Figure adapted from [89].

Voltage‐gated calcium (Ca_V_) channels are involved in a multitude of cellular responses, including muscle contraction, neuronal activation, and neurotransmitter release [84, 85, 86]. Three classes of Ca_V_ channels have been identified; these are responsive to high voltages (L, N, P/Q, R Ca_V_'s) generated by large membrane de‐polarizations or to low voltages (T‐type Ca_V_'s) [87]. Five types of Ca_V_ channels can be differentiated on the basis of their electrophysiological characteristics and their α1‐sub‐units; these are grouped into pharmacologically distinct Ca_V_ channel subtypes, including the L‐, N‐, T‐, R‐, and P/Q‐type calcium channels. Ten isoforms have been identified [88] (Figure 3). N‐, T‐, and P‐type Ca_V_ channels have been targeted for the alleviation of pain [90, 91, 92, 93]. N‐type (Ca_v_2.2) calcium channels are transmembrane high‐voltage activated Ca channels that are concentrated in neurons and have roles in the processing of sensory pain [89]. Ca_V_2.2 is highly concentrated in spinal dorsal horn neurons, DRG cell bodies, and spinal dorsal horn neuron synaptic connections. N‐type Ca_V_ channels have major roles in the initiation and maintenance of pain following peripheral nerve damage and thus represent therapeutic targets to block nociceptive processes of pain generation [94, 95]. The Ca_V_2 subfamily of Ca^2+^ channels is insensitive to dihydropyridine Ca^2+^ channel blocker drugs but is blocked with high affinity by spider and marine snail peptide toxins with high specificity [96]. Ca_V_2.1 channels are blocked specifically by funnel web spider venom ω‐agatoxin IVA; Ca_V_2.2 channels are blocked by cone snail ω‐conotoxin GVIA toxins. The Ca_V_2.3 channel family is blocked specifically by the synthetic peptide toxin SNX‐482 derived from tarantula venom, blocking synaptic transmission and pain generation.

The Ca_V_3 T‐type calcium channels differ from the other Ca channels, being responsive to tiny and transient currents generated during membrane depolarization. Furthermore, unlike Ca_V_1 and Ca_V_2, the T‐type Ca_V_ channels do not require interactions with multiple auxiliary subunits for their Ca^2+^ channeling activities [89, 97, 98, 99, 100, 101]. A large degree of sequence variations in the T type channels compared to high voltage‐gated Ca^2+^ channels has been observed, supporting their independent functional properties [99, 100, 102]. Ca_V_3.2 is widely expressed in the CNS and regulates neuronal excitability and has roles in nociception [103, 104]. Abnormal Ca^2+^ channeling is implicated in neurological and neuropsychiatric disorders, epilepsy, and pain [105]. Ca_V_3.2 expression is elevated in spinal horn and DRG neurons in models of neuroinflammation and NeP [105]. Silencing of Ca_v_3.2 channel activity induces analgesia in rodents [106].

The Ca_V_3 subfamily of Ca^2+^ channels is relatively insensitive to Ca_V_1 blocking dihydropyridine‐based compounds and the Ca_V_2 spider and cone snail toxin blocking peptides [107], mibefradil is a selective blocker of T and L‐type Ca^2+^ currents (3‐ to 10‐fold). The peptide kurtoxin, from the venom of the scorpion *Parabuthus transvaalicus*, also inhibits the activation of Ca_V_3.1 and Ca_V_3.2 channels [108]. A few recently developed pharmacological agents (TTA‐A2, TTA‐P2, and Z944) selectively block T‐type calcium channels in models of NeP, epilepsy, and psychiatric comorbidities [107, 109]. Z944 is also effective in NeP in humans; cryo‐EM structures of human Ca_v_3.1 and in complex with Z944 have been published [110].

### 6.2 Blocking of Neuronal Processes Using Ca Channel Blocking Peptides

#### 6.2.1 Therapeutic Anti‐Nociceptive Peptides of Reptilian and Insect Venoms

Neurotoxins are of considerable interest in neurobiology and can potentially provide a better understanding of mechanisms involved in neurotransmission and pain [88]. While snakes, spiders [111, 112], and cone snails produce venoms with toxic effects, they also contain a wide range of neuropeptides with useful antinociceptive properties [113]. Antinociceptive peptides have also been identified in wasp venom [114] and skin secretions of some amphibians [115]. Table 1 provides a comprehensive overview of calcium channel subtypes, their localization, physiological functions, and specific blocking peptides, illustrating their diverse roles in cellular processes and therapeutic potential.

**TABLE 1:** Calcium channel blocking agents.

Ca^2+^ channel	Ca^2+^ channel type	Specific blocking agent	Localisation	Function	References
Ca_V_1.1	L	Dihydropyridine, nifenidine, amiodipine, verapamil, diltiazem, Black mamba toxin Calciseptine, ω‐agatoxin IIIA (α1C), calcicludine	Exclusively located in skeletal muscle	Skeletal muscle contraction, regulation of Ca^2+^ homeostasis, electrophysiological conductance kinetics atypical of the other Ca_V_1 channels	[116, 117, 118, 119, 120]
Ca_V_1.2	L	Dihydropyridine, nifenidine, amiodipine, verapamil, diltiazem, Phα1β, Black mamba toxin Calciseptine, ω‐agatoxin IIIA (α1C), calcicludine	Cardiac muscle, endocrine cells, neurons	Regulation of cardiac muscle, neuronal activity and hormonal release by endocrine system	
Ca_V_1.3	L	Dihydropyridine, nifenidine, amiodipine, verapamil, diltiazem. Black mamba toxin Calciseptine, ω‐agatoxin IIIA (α1C), calcicludine	Endocrine cells, neurons	Regulation of endocrine cells and neuronal activity, control of hormone secretion	
Ca_V_1.4	L	Dihydropyridine, nifenidine, amiodipine, verapamil, diltiazem, Black mamba toxin Calciseptine, ω‐agatoxin IIIA (α1C), calcicludine	Photoreceptors in retina	Required for normal vision, regulates neurotransmitter release and phototransduction to bipolar neurons	
Ca_V_2.1	P/Q	ω‐Agatoxin MVIIC, Phα1β^a^, CΤΚ 01512–2	Exclusively in neuronal dendritic nerve terminals	Pain alleviation, regulation of neurotransmitter release and neurotransduction	[84, 85, 89, 90, 92, 94, 95, 115, 117, 121, 122, 123, 124, 125, 126, 127, 128, 129, 130]
Ca_V_2.2	N	ω‐Conotoxin, ω‐GVIA, ω‐MVIIA, (ω‐CVI)D, Phα1β^a^, CΤΚ 01512–2, zixonotide	Exclusively in neuronal dendritic nerve terminals	Pain alleviation, blockade of Ca_V_ 2.2 inhibits nociceptor pain generation, Phα1β blocks > 95% Ca_V_2.2 channel transmissions	
Ca_V_2.3	R	SNX‐482 Tarantula toxin, Phα1β^a^, CΤΚ 01512–2	Neuron cell bodies, dendrite nerve terminals	Regulation of Ca^2+^ dependent action potentials and neuronal activity	
Ca_V_3.1	T	NNC‐55‐0396, Kurtoxin (α1G) from scorpion *Parabuthus transvaalicus*, TTA‐A2, TTA‐P2, Z944	Cardiac and skeletal muscle, neurons	NNC‐55‐0396 selective T channel blocker, IC_50_ value of 6.8 μM for Cav3.1, Control of neuronal excitability by small membrane depolarization, pacemaker repeated neuronal signaling	[87, 89, 91, 100, 103, 104, 105, 107, 108, 109, 131]
Ca_V_3.2	T	NNC‐55‐0396, Mibefradil, Kurtoxin (α1G) from scorpion *Parabuthus transvaalicus*, TTA‐A2, TTA‐P2, Z944	Cardiac muscle, neurons	Abnormal Ca channeling is implicated in neurological and neuropsychiatric disorders, epilepsy and pain. Control of neuronal excitability by small membrane depolarization, pacemaker repeated neuronal signaling	
Ca_V_3.3	T	Mibefradil, Kurtoxin (α1G) from scorpion *Parabuthus transvaalicus*, TTA‐A2, TTA‐P2, and Z944	neurons	Control of neuronal excitability by small membrane depolarization, pacemaker repeated neuronal signaling	

^a^ Phα1β Ca^2+^ channel blocker potencies *N* > *R* > P/Q > L, also acts as a TRPA1 channel antagonist, CΤΚ 01512–2 re‐ombination Phα1 peptide.

Several venom‐derived peptides have shown efficacy in preclinical models of NeP. ω‐Conotoxin MVIIA (ziconotide), derived from *Conus magus*, has demonstrated robust analgesic effects in spinal nerve ligation and chronic constriction injury models [95, 132]. Similarly, *Phoneutria nigriventer* peptide Tx3‐5 has shown significant antinociceptive activity in NeP models, reducing mechanical and thermal hypersensitivity [133]. These findings suggest that specific venom peptides may target calcium channels and other ion channel subtypes involved in NeP pathways, providing a strong rationale for their investigation in discogenic LBP where both nociceptive and neuropathic components may coexist.

#### 6.2.2 Toxins That Specifically Target Neuronal Responses

Nigriventer venom peptides such as PnTx3‐6 have a strong antinociceptive effect in cancer‐related pain and have minimal side effects even at high doses. Nigriventer peptides are more effective and potent antinociceptive agents than ω‐conotoxin peptides isolated from cone snail venom that inhibit Na_v_ channels with great potency and selectivity [133, 134, 135, 136].

#### 6.2.3 Conotoxins

The venom of the marine predatory cone snails (genus *Conus*) contains hundreds to thousands of bioactive peptides known as conotoxins. These are disulfide‐rich, well‐structured peptides which act on a wide range of targets such as ion channels, G protein‐coupled receptors, transporters, and enzymes. Conotoxins are of interest to neuroscientists as well as in drug development due to their exquisite potency and selectivity and potential therapeutic applications in the alleviation of pain. Over 10 000 conotoxin sequences have been published; however, data on their structure and pharmacology is incomplete. Conotoxin peptides interact with the nervous system, disrupting the activity of ion channels, including sodium, calcium, and potassium channels involved in the transmission of pain signals [137, 138, 139]. ω‐conotoxins identified in the venom of cone snails are basic peptides of 24–29 residues with an amidated *C*‐terminus, with six Cys‐residues arranged to form a Ca^2+^ interactive Cys framework (C–C–CC–C–C). Most of the ω‐conotoxins selectively inhibit N‐type Ca_V_ channels; a few also target the P/Q‐type Ca_V_ channel. Conotoxins ω − GVIA and ω − MVIIA are the most extensively studied of the cone peptides [140, 141]. ω‐Conotoxins GVIA, MVIIA, and CVID, selective N‐type Ca_V_ channel blockers, are neuroprotective and also possess analgesic properties [121, 122, 123, 124, 139] thus, they are of interest in drug development. Many of the other conotoxin peptide inhibitors, however, appear to have toxic profiles in mammals. The ω‐conotoxin MVIIA, derived from the venom of *Conus magus*, is marketed as Prialt (ziconotide). Ziconotide has been approved by the FDA to treat chronic pain as an alternative to opioid analgesics, which have been problematic due to their addictive properties [125, 126, 142, 143].

#### 6.2.4 Brazilian Wandering Spider *(Phoneutria nigriventer)* Toxins

*Nigriventer* venom toxins act on sodium, calcium, and TRPA channels, and cannabinoid or opioid receptors [144, 145, 146]. *P. nigriventer* venom contains at least six bioactive peptides collectively referred to as PhTx3 (Tx3‐1 to Tx3‐6) [147]; Tx3‐3 is also known as ω‐ *Phoneutria nigriventer* toxin ω‐PnTx3‐3 [148] and Tx3‐4, phonetoxin IIA or ω‐Ptx‐IIA [149]. These toxins act as broad‐spectrum calcium channel blockers that inhibit glutamate release and calcium and glutamate uptake in synaptosomes. A synaptosome is an isolated portion of a neuronal synaptic terminus containing the morphological features and chemical properties of the synaptic nerve terminal. Synaptosomes are used to study synaptic transmission in vitro since they contain the molecular machinery involved in the uptake, storage, and release of neurotransmitters and are thus a useful tool for drug testing. Two PhTx3 isoforms, Tx3‐3 and Tx3‐4, effectively inhibit the influx of Ca^2+^ in rat cortical synaptosomes induced by the scorpion venom tityustoxin, displaying IC50 values of 0.32 and 7.9 nM, respectively; these maintain low nanomolar intrasynaptosomal calcium levels.

A PnPP‐19 peptide, representing a discontinuous epitope in PnTx2‐6, has been synthesized to circumvent unwanted toxic side effects and has central and peripheral antinociceptive activity involving the opioid and cannabinoid systems. Pharmacological inhibition of voltage‐gated Ca^2+^ channels has the potential to provide chronic pain relief [150]. Regional nerve blocks have been used to alleviate pain in hip fractures [151]. Studies are on‐going in the development of pain inhibitors that potentially may be applicable in the treatment of discogenic LBP [152, 153, 154, 155]. Of the *Nigriventer* venom peptides, PnTx2‐6 is one of the most potent calcium‐blocking anti‐nociceptive peptides [127]. Tx3‐3 and Tx3‐5 have also been examined in animal models of chronic pain, and both are reported to have beneficial properties [128, 133]. Tx3‐3, Tx3‐4, and Tx3‐6 all display potent Ca‐blocking anti‐nociceptive properties [127, 148, 156, 157]. Recombinant *Phoneutria nigriventer* venom peptides inhibit nociception by nerve deafferentation [158]. Deafferentation is the interruption or destruction of the afferent connections of nerve cells [152]. Phoneutria toxin PnTx3‐5 also inhibits TRPV1 channels and has antinociceptive action in an orofacial pain model [153]. Collective evidence thus points to the potential application of these toxins in the treatment of discogenic LBP. Rather than the administration of these peptides directly on nerve tissues or DRGs, some of these peptides, such as PnPP‐19, may be suitable for incorporation as bioactive modules in biomimetic PGs, where they may serve as pro‐drugs that are released when IVDs remodel during degenerative processes, ensuring such peptides are released in an active form at sites where nerve ingrowth may occur, ensuring specificity of action. Phα1β and its recombinant form CTK 01512–2 are peptide toxins that have satisfactory safety profiles.

Ca_v_ channels convert the membrane generated electrical signals into intracellular Ca^2+^‐mediated events. Ca_v_2.2 (N‐type) are high‐voltage‐activated channels, with auxiliary subunits, such as extracellular α2δ, intracellular β, and the transmembrane γ [116]. The intracellular I–II and III–IV linker helices interact with the β1α‐subunit and the carboxy‐terminal domain of α1, respectively. These channels are distinguished by being exclusively distributed in neuronal tissue, mainly in the synaptic nerve terminals in laminae 1 and 2 calcium channels, namely the L‐(Ca_v_1.2), N‐(Ca_v_2.2), P/Q‐(Ca_v_2.1), and R‐(Ca_v_2.3) types, with different potencies (N > R > P/Q > L) [127] and TRPA1 [117]. The action of both the native and recombinant toxins is comparable [125, 128, 129, 130, 131, 132, 159].

#### 6.2.5 Other Venom Peptides That Have Been Evaluated as Calcium Blocking Agents Active in the Alleviation of Pain in Animal Models

The black mamba ( *Dendroaspis polylepis* ) toxin Calciseptine displays Ca channel blocking activity and pain alleviating properties [118, 119]. Scorpion venom also contains a number of toxin peptides that have pain alleviating activities exerted through transient receptor potential channels and ATP sensitive receptors [111]. The most potent venom peptides have been characterized from *Pandinus imperator, Chari‐lustricostatus, Buthus martensii, Mesobuthus eupeus, Leiurus quinquestriatus, Tityus discrepans, Heterometrus bengalensis with* over 1500 species of scorpions identified globally [160]. At least 800 disulfide stabilized multifunctional bioactive venom peptides have been characterized including buthitoxin Ctriporin, Chlorotoxins, Neopladine I and II, Meucin 24, Meucin 25, iberiotoxin, and Hp 1090 [161, 162, 163]. Some of these have therapeutic biomedical potential [120, 164, 165, 166, 167].

### 6.3 Mechanistic Link Between Venom Peptides, Calcium Channels and Analgesia

Venom‐derived peptides achieve robust analgesia by targeting VGCCs, particularly the N‐type Ca_V_2.2 channels that orchestrate activity‐dependent neurotransmitter release from DRG afferents in the spinal dorsal horn [154, 166]. ω‐Conotoxins such as MVIIA and CVID bind with nanomolar affinity to the external vestibule of the Ca_V_2.2 α1B subunit, producing a noncompetitive steric block that prevents channel opening during neuronal depolarization. The resulting suppression of calcium‐dependent exocytosis of glutamate and substance P interrupts the first synapse of the pain pathway and curtails central sensitization.

Selectivity is a key advantage: MVIIA and CVID largely spare L‐ and P/Q‐type channels, minimizing off‐target cardiovascular and cognitive effects. Structure–activity studies attribute this precision to the sequence and spacing of intercysteine loops; by contrast, the spider peptide Phα1β, whose loop chemistry differs, blocks both N and P/Q‐type channels and therefore shows a broader analgesic spectrum [168]. Such molecular fingerprints translate into distinct in vivo profiles: intrathecal MVIIA reverses mechanical allodynia within minutes in spinal nerve ligation models, whereas systemic Phα1β attenuates paclitaxel‐induced hypersensitivity without motor impairment [154, 168].

NeP frequently features upregulated Ca_V_2.2 expression and enhanced synaptic calcium currents in injured DRG neurons, changes that amplify spontaneous firing and dorsal horn hyperexcitability. Venom peptides therefore act upstream of this maladaptive plasticity. PnTx36 from *Phoneutria nigriventer* , for instance, inhibits Ca_V_2.2 and auxiliary channel subtypes, reducing both mechanical allodynia and thermal hyperalgesia in diabetic and inflammatory neuropathy models [133].

Therapeutic development now focuses on improving stability and pharmacokinetics. CVID (AM336) has demonstrated Phase II efficacy, while engineered αconotoxins and conantokins expand the modality by targeting complementary nodes such as nicotinic acetylcholine and NMDA receptors [166]. Highthroughput “metavenome” phagedisplay platforms further accelerate discovery, screening millions of disulfide‐rich scaffolds to identify next‐generation Ca_V_2.2 inhibitors with enhanced serum stability and diminished neuropsychiatric liability [142].

Collectively, these findings establish a direct, mechanistic bridge from venom peptide binding at Ca_V_ channels through synaptic inhibition to durable reversal of nociceptive and NeP behaviors.

## 7 Current Downfalls and Future Clinical Implications

Many conservative therapies currently fail to adequately treat chronic LBP due to the multifaceted nature of its underlying pathophysiology. Existing conservative injections, primarily steroid‐based, are designed to target perineural inflammation; however, intraoperative observations during lumbar decompression have shown that not all patients exhibit inflammatory changes, and the overall clinical effectiveness of these treatments remains a topic of ongoing debate. Therefore, innovative therapeutic approaches are urgently needed that go beyond inflammation alone.

Given the involvement of voltage‐gated calcium channels in NeP signaling, the ability of venom‐derived peptides to inhibit these pathways in established neuropathic models further supports their potential clinical application. Targeting both nociceptive and neuropathic mechanisms may provide a more comprehensive approach to managing chronic LBP.

One promising direction, as explored in this review, involves targeting calcium channels using naturally occurring venom‐derived peptides. Previous intradiscal analgesic therapies generally fall into two categories: short‐term pain relief approaches, such as corticosteroid injections, and long‐term regenerative strategies, such as the use of Growth Differentiation Factor 6 (GDF6) to promote IVD regeneration [169]. The venom‐derived peptides discussed here could potentially be combined with biomimetic proteoglycans or regenerative biologics, providing a dual approach that delivers both immediate nociceptive pain relief and longer‐term functional recovery by addressing structural degeneration [83].

Importantly, both nociceptive and NeP pathways are ultimately mediated through neural circuits originating in the DRG. As such, these venom‐derived calcium channel blockers may exert their therapeutic effects by inhibiting DRG signaling and interrupting pain transmission. Targeting the disc directly—via intradiscal injections—may further provide a localized approach to reducing nociceptive pain arising from degenerated disc tissue.

We acknowledge the substantial challenges associated with systemic delivery of calcium channel blockers, as exemplified by agents like ziconotide (Prialt), which require intrathecal administration due to severe systemic effects [170]. However, the spine offers unique opportunities for localized delivery. In particular, intradiscal and facet joint injections could serve as targeted delivery routes, with the facet joint potentially functioning as a local drug reservoir, facilitating extended drug release into the surrounding tissues. These targeted approaches may help mitigate the risks of systemic toxicity while maximizing local efficacy. Future preclinical and clinical studies will be essential to determine the optimal delivery strategies—whether via epidural, facet, or intradiscal injections—and to explore potential synergies with regenerative therapies or surgical interventions.

## 8 Conclusions

This review has presented the potential of targeting the degenerative IVD and the DRG for pain blocking and the therapeutic benefits of naturally occurring bioactive peptides. Anti‐nociceptive peptides that occur in reptilian and insect venoms are potential candidates for incorporation into current IVD regeneration and nerve block procedures, which may potentially provide pain alleviating properties. A review of these venom peptides uncovered several candidate peptides with potent blocking properties over voltage‐gated calcium channels, preventing neuronal activation, neurotransduction, and nociception. These blocking agents may be suitable for direct therapeutic application to IVDs or DRGs and offer exciting possibilities in pain alleviation.

## Conflicts of Interest

S.S. was supported by an Australian Government's University Postgraduate Award from the University of New South Wales. All authors declare that they have no known competing financial interests or personal relationships that could have appeared to influence the work reported in this paper. Spine Labs is supported via unrestricted research grants to its institution by Baxter Inc.
